# Development of a Polyherbal Topical Gel for the Treatment of Acne

**DOI:** 10.3390/gels9020163

**Published:** 2023-02-17

**Authors:** Benedict Jose Chellathurai, Ramyadevi Anburose, Mohammad H. Alyami, Mohan Sellappan, Mohammad F. Bayan, Balakumar Chandrasekaran, Kumarappan Chidambaram, Mohamed Rahamathulla

**Affiliations:** 1 Department of Pharmaceutics, Karpagam College of Pharmacy, Coimbatore 641 032, Tamil Nadu, India; 2 Department of Pharmaceutics, College of Pharmacy, Najran University, Najran 66462, Saudi Arabia; 3 Faculty of Pharmacy, Philadelphia University, P.O. Box 1, Amman 19392, Jordan; 4 Department of Pharmacology, College of Pharmacy, King Khalid University, Abha 62529, Saudi Arabia; 5 Department of Pharmaceutics, College of Pharmacy, King Khalid University, Abha 62421, Saudi Arabia

**Keywords:** *Aloe barbadensis*, *Vigna radiata*, Carbopol 940, polyherbal gel, acne

## Abstract

The present work aimed to formulate and evaluate a polyherbal gel using *Aloe barbadensis* and extract of *Vigna radiata* for the treatment of acne, a disorder of the skin in which hair follicles and sebaceous glands are blocked, causing inflammation and redness of the skin. *Aloe barbadensis* pulp was collected and mixed with the extract of *Vigna radiata* and formulated into a gel using Carbopol 940, triethanolamine, and propylene glycol as the gelling agent, viscosity modifier, and pH modifier, respectively. The gel was evaluated for its antimicrobial properties against *Staphylococcus aureus*, *Escherichia coli*, and *Candida albicans*. Antimicrobial agents, such as gentamycin and fluconazole, were used as the standards. The developed formulation showed promising zone of inhibition. The gel was further evaluated for its physicochemical properties. The formulation showed a promising effect on acne together with the additive effect of *Aloe barbadensis* on skin.

## 1. Introduction

Since the beginning of time, humans have employed natural herbals in their life to treat illness and to support a healthy lifestyle. Natural phytoconstituent-based formulations have gained universal acceptance as therapeutic agents for the treatment of inflammatory conditions, infections, arthritis, hyperglycemia, depression, anxiety, HIV, and other diseases [1]. Increased attention has been focused on the development of polyherbal formulations because of their traditional background, cost effectiveness, and patient compliance. Currently, there are numerous treatments available that employ topical, biological, and systemic medicines. Some of the medications assist in lessening the symptoms of diseases, but they also have some adverse effects. The development of a medication with high efficacy and few adverse effects is crucial in the interim. Herbal medications are safer and more effective in reducing symptoms than allopathic ones [2]. Any plant that includes compounds with therapeutic properties or compounds that can be utilized as building blocks for semi-synthetic pharmaceuticals is considered a medicinal plant. These phytochemicals, which are non-nutrients found in plants, act as defense mechanisms for the plants against microbial infections. Acne, a skin disorder, develops when dead skin cells clog hair follicles. Acne vulgaris, which is defined by the development of inflammatory and non-inflammatory lesions of hair follicles and sebaceous glands, affects approximately three-fourths of individuals in the age range of 11 to 25. Acne may be brought on by hormonal imbalance, environmental conditions, or a hereditary predisposition that results in the development of comedones. When bacteria enter a blocked pore, they produce chemicals that trigger inflammation and result in the development of pimples and pustules. Severe inflammation creates nodules, which develop into cysts and, once cured, may leave scars [3]. Gels are employed to ensure the best possible cutaneous and percutaneous medication delivery. They can prevent gastrointestinal medicine-absorption issues brought on by acidic gastrointestinal conditions. Gels have the ability to prevent medication interactions with food and drink, as well as enzymatic activity. When the oral route is inappropriate, they may be used in place of oral administration of medicines. They can avoid the first pass effect, and due to the liver’s bypass, gels are not inactivated by degrading enzymes. Patients comply with gel formulations as they are non-invasive. Gels have also been used to dispense some viscous oral suspensions, such as aluminum hydroxide gel. When compared to creams and ointments, topical administration of gels at pathological locations has significant advantages in terms of direct drug release and speedier absorption. In skin care products, single-phase gel is widely used. Organic macromolecules are uniformly disseminated throughout a liquid in such a way that there are no obvious boundaries between the dispersed macromolecules and the liquid. This makes them ideal for beauty goods due to their appealing appearance. As it is intended for use on big hairy regions, such as the chest and back, an optimal acne formulation for those areas should distribute smoothly and leave no residue or oiliness [4]. Given its superior viscosity-building capabilities (even at low concentrations) and resistance to microbial development, Carbopol^®^ 940 is commonly utilized in topical formulations. The therapeutic approach to treating acne is one of the more promising areas since it must overcome the psychological impact on patients. A safe method for treating skin infections is through topical medication delivery. Antibiotics and anti-inflammatory medications can be used orally or topically as part of current acne treatment. For mild and moderate acne, topical therapy is the first line of defense. Antibiotics, acids, benzoyl peroxide, retinoids, herbal agents, or a combination of topical medications are the basis of topical therapies. Although topical therapies are less dangerous than systemic ones, they often have low water solubility and insufficient free drug penetration across the stratum corneum. However, systemic therapy is also necessary for moderate to severe cases [5,6]. The limitations of topical therapies may be circumvented by encasing or conjugating these compounds with nanocarriers. It is feasible to categorize distinct nanocarriers according to their nature. Organic nanocarriers, such as liposomes, nano emulsions, and dendrimers, are preferred to inorganic nanocarriers, such as metallic nanoparticles, because they may be altered in terms of their chemical makeup, surface, shape, and size, thereby changing their performance and attributes. Herbal-based nano formulations show notably improved characteristics, including controlled release, targeted distribution, and greater drug-loading capacity [7]. To achieve better results, several traditional approaches have been used [8,9,10]. Antimicrobial agents have been used for a long time, which has caused the bacteria that cause acne, i.e., Propionibacterium acne and *Staphylococcus epidermis* [11]. *Vigna radiata* is one of the most significant summer-growing, short-season legumes. It contains a variety of chemical components that are renowned for their antioxidant, antibacterial, and anti-inflammatory properties, including flavonoids, phenolic acids, and organic acids. *Aloe barbadensis* is a crucial ingredient in cosmetics. Aloe gel is preferred when formulating various topical medications for skin conditions, such as burns, wounds, acne, rashes, psoriasis, cold sores, or dry skin. It is also used as a carrier and an emollient to treat burns, pigmentation, acne, and other skin disorders [12]. The main aim of this study was to formulate the aforementioned herbal constituents into an efficient and safe topical dose in the form of a gel. The prepared formulations were assessed for their physical characteristics.

## 2. Results and Discussion

### 2.1. Organoleptic Evaluation of the Extract of Vigna radiata

Organoleptic evaluation is a qualitative method in which researchers evaluate the distinct qualities of drugs, particularly those with a plant origin, by using their senses (sight, smell, taste, hearing, and feeling) while recording information about the size, shape, colors, marks, fractures, textures, odors, and tastes. The methodology used in this study is often referred to as organoleptic, and the data collected are known as sensory characteristics. The physical state, color, odor, and taste of the ethanolic extract of *Vigna radiata* are presented in Table 1, which indicate that the extract of *Vigna radiata* possesses its typical characteristics [13].

### 2.2. Phytochemical Investigation of the Extract

Phytochemical investigation is important since different classes of phytoconstituents that are found in various drug bases are analyzed, extracted, and identified. Phytochemical screening not only assists in identifying the components of plant extracts and which component predominates over the others, but it also aids in the search for bioactive substances that can be employed in the synthesis of therapeutic medications. Alkaloids, flavonoids, phenolic compounds, saponins, steroids, tannins, and terpenoids are just a few of the substances that are significant for both industrial and therapeutic purposes and can be found in novel sources through phytochemical screening. The phytochemical investigation of the extract of *Vigna radiata* revealed the presence of flavonoids, phenolics, and other constituents. Many of these compounds have been shown to produce potent antimicrobial, antidiabetic, and antihyperlipidemic activities. The results are presented in Table 2.

### 2.3. Antimicrobial Activity of the Extract

**Figure 1:**
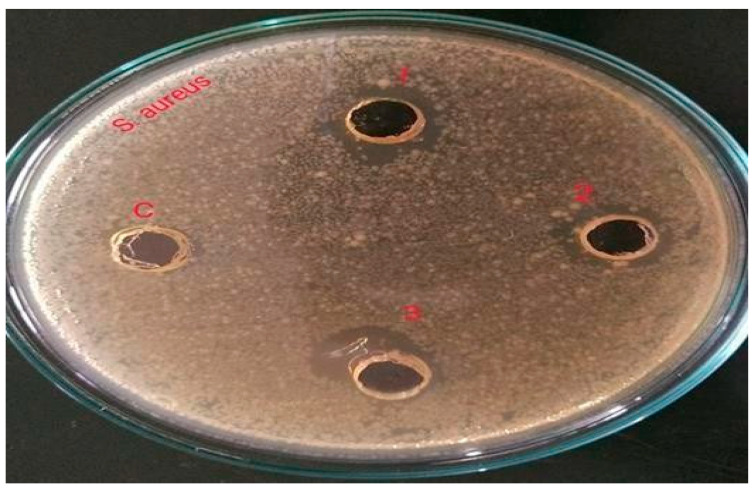
Zone of inhibition of *Vigna radiata* extract towards *Staphylococcus aureus* (**1**) 12.4 mm, (**2**) 12.3 mm, (**3**) 12.1 mm and (**c**) control.

**Figure 2:**
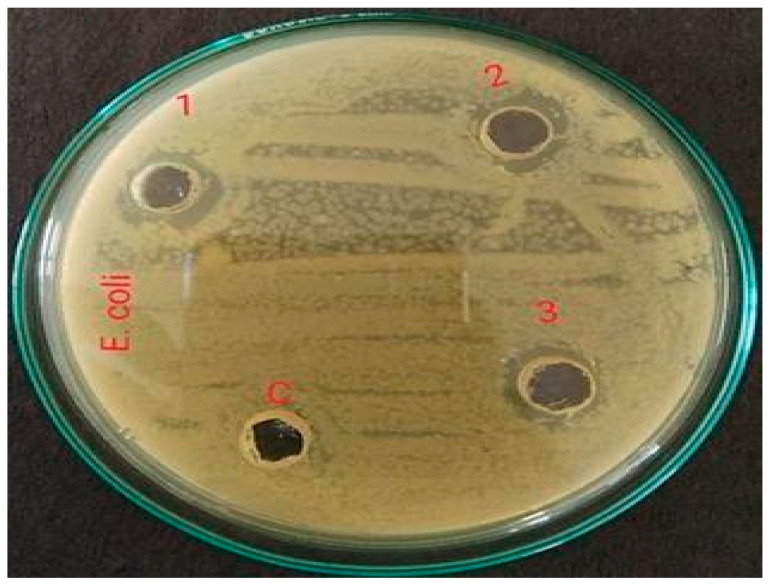
Zone of inhibition of *Vigna radiata* extract toward *Escherichia coli* (**1**) 13.2 mm, (**2**) 13.1 mm, (**3**) 13.1 mm and (**c**) control.

**Figure 3:**
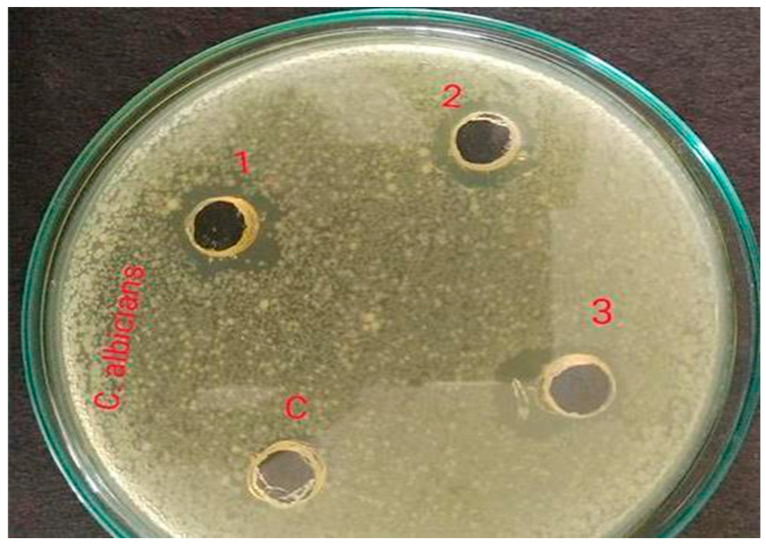
Zone of inhibition of *Vigna radiata* extract toward *Candida albicans* (**1**) 12.4 mm, (**2**) 12.3 mm, (**3**) 12.5 mm and (**c**) control.

**Figure 4:**
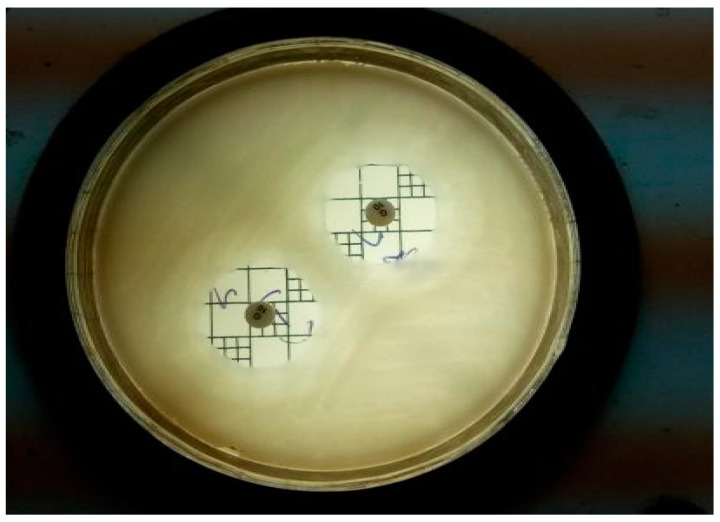
Standard Gentamycin on *Staphylococcus aureus* at 10 mcg in duplicate at ZOI of 22.2 mm.

**Figure 5:**
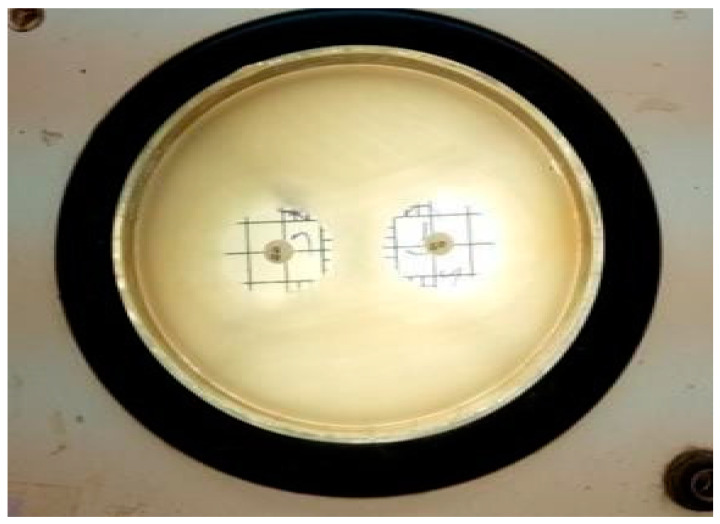
Standard Gentamycin on *Escherichia coli* at 10 mcg in duplicate at ZOI of 22.3 mm.

**Figure 6:**
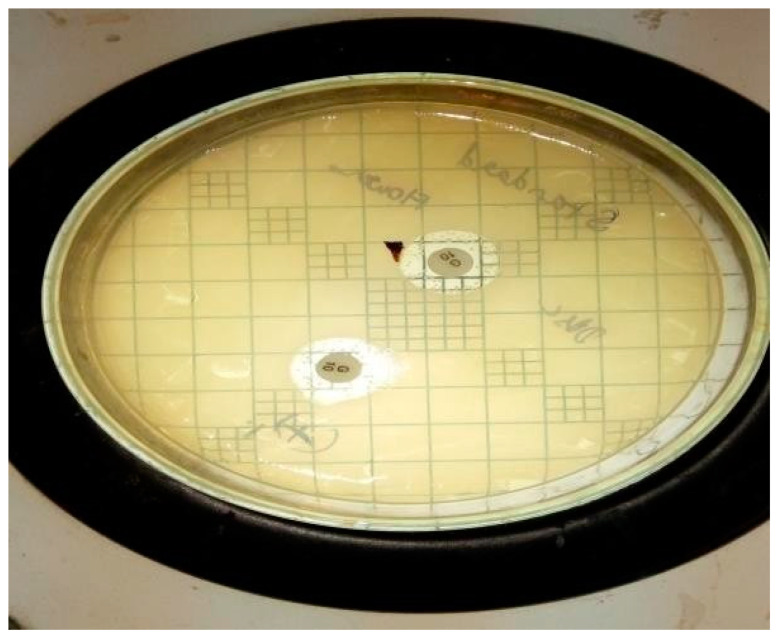
Standard Fluconazole on *Candida albicans* at 25 mcg in duplicate at ZOI of 22.2 mm.

The antimicrobial activity testing was performed by relating the diameter of zones of inhibition (in mm), which indicates the effectiveness of an antimicrobial agent. The extract of *Vigna radiata* was observed for its antimicrobial property toward acne-causing organisms, such as *Staphylococcus aureus* (ATCC-6538P), *Escherichia coli* (ATCC-8739), and *Candida albicans* (ATCC-18804). Its activity was also compared with the standards, such as Gentamicin (10-mcg) and Fluconazole (25-mcg). It is believed that the antimicrobial property might be due to the presence of strong flavonoids in the extract and the weak antioxidant nature of the gel, which in turn increases the shelf life of the product from photodegradation and oxidative degradation. The antimicrobial property of the gel might be due to the high percentage content of flavonoids, which makes the preparation highly effective against the studied microorganisms. Moreover, the content of polyphenols present in the extract exhibits anti-acne activity due to the opening of mitochondrial permeability transition pore (mPTP) [14]. Additionally, the antioxidant property that protects epidermal cells from UVA-induced damage is mainly due to the presence of orthophosphoric acid in the extract, which protects the skin from conditions of extreme pH modification [15]. From the results, it was observed that the prepared gel showed a good zone of inhibition (around 12.5 mm), although smaller when compared to the standards (around 22 mm); the results are shown in Table 3 and Figure 1, Figure 2, Figure 3, Figure 4, Figure 5 and Figure 6.

### 2.4. Optimization of Gelling Agent

A gel is usually made up of two components: a hydrophilic polymer and water, alcohol, or another solvent (such propylene glycol). The hydrophilic polymer serves as a gelling agent in the majority of gels, which employs water. There are several distinct types of gelling agents that can be used. With respect to the amount of gelling agent utilized, the main property is the viscosity they offer. Those that produce a harder gel, however, frequently make it more brittle and prone to collapsing. There are certain incompatibilities as well, with some working better at specific pH levels than others. The gelling agent and the solubilizer are the crucial components in the formulation of a gel. A gel can be clear, translucent, or opaque depending on the excipients employed. Choosing a proper gelling agent and maintaining a drug’s solubilized state are challenging aspects of the formulation process. Different concentrations of Carbopol 940, including 1, 1.5, and 2%, were optimized to obtain the gel with the desired physical characteristics. The Carbopol gel with 2% concentration (G3) showed good physicochemical properties for incorporating ethanolic extracts of *Vigna radiata* and *Aloe barbadensis*. Table 4 presents the constituents of each formula.

### 2.5. Formulation of Polyherbal Gel Containing Vigna radiata and Aloe barbadensis

Plants are regarded as an important source of potentially beneficial components for the creation of novel therapeutic medicines because the majority of them are harmless and have few or no side effects. Comparing topical gels to cream or ointment application, gels offer tremendous advantages in terms of a faster release of the medicine directly to the site of action. The topical administration of medications is now frequently carried out using gels. This dosage form might include extracts of plants and herbs with particular therapeutic qualities as active ingredients to provide additional advantages. The polyherbal gel containing *Vigna radiata* and *Aloe barbadensis* were incorporated into the optimized 2% Carbopol gel base. Different concentrations of ethanolic extract of *Vigna radiata*, including 1,1.5, and 2%, were also incorporated into the gel base. The *Aloe barbadensis* concentration was kept constant [5 mL] in all the gel base. The formulations of the designed polyherbal gel are presented in Table 5.

### 2.6. Evaluation of Polyherbal Gel Physical Appearance

**Figure 7:**
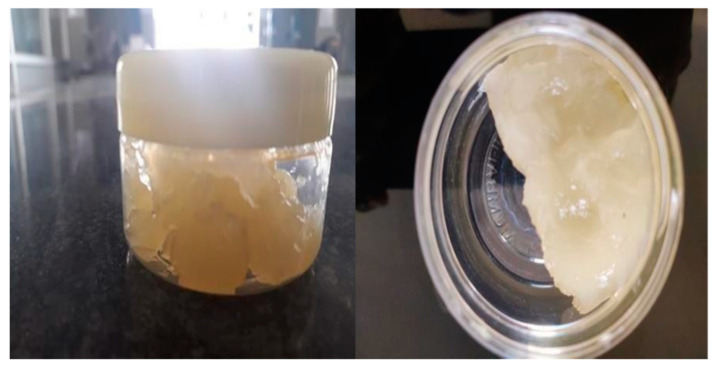
Image of formulated polyherbal gel containing *Vigna radiata* and *Aloe barbadensis*.

The formulated gel was checked visually for color, appearance, and homogeneity; the results are listed in Table 6 and Figure 7, which indicate the absence of aggregates with F1 and F2, and the presence of slight aggregates in F3 (2% *Vigna radiata* extract). This indicates a problem in homogeneity in F3.

### 2.7. pH Determination

A good topical preparation should have a pH that is acceptable for the skin, ranging from 4.2 to 6.5. Gels that are too alkaline will result in scaly skin. On the other side, if the pH is too acidic, it will irritate the skin. The pH of the formulation was 5.7–5.9. The pH of the prepared gel showed its compatibility with the skin. Even though the ideal pH ranges below 5.0, the addition of stabilizers contributes to this pH range, making it suitable for topical application and penetration [16].

### 2.8. Determination of Viscosity

Rheological characteristics of gels vary and show reversible deformation, similar to that experienced by elastic materials, rather than flowing at low shear stresses. They flow like liquids when a specific shear stress is exceeded, which is known as the yield value or yield stress. In general, the consistency of gel compositions is reflected in their viscosity. Non-Newtonian flow (shear thinning) shows how the viscosity of gels reduces with increasing shear rate; this behavior is desired because of its low flow resistance when used under high shear conditions. The rheological property helps in determining consistency and influence the diffusion rate of a drug from a gel. By maintaining the viscosity below 15,000 cps, the advantages of more attractive cosmetic characteristics and ease of accurate application over the skin through better flow and spreadability can be achieved. Additionally, this low viscosity is an indication of the viscoelastic behavior of the gel upon applied stress, which makes it easier to flow from the container to the applying area and suck back to the container upon the release of stress [17]. The results are tabulated in Table 7. It can be observed that all formulations have low viscosity, which indicates promising applicability for skin administration. 

### 2.9. Spreadability

Manufactured gels must have good spreadability and satisfy the ideal quality in topical application since the spreadability of the gel aids in the uniform application of the gel to the skin. In addition, it is thought that this is a key element in patient adherence to therapy. Spreadability denotes the area and the extent to which a gel readily spreads upon topical application [18]. The spreadability of the different gel formulation were studied. The formulation F2 produced better spreadability than the other formulations. The results of the three physical parameters are presented in Table 7. To have a good permeation across the skin, the gel should have ideal property and stability over a long period. From the results obtained for the physical parameters, such as pH, viscosity, and spreadability, it can be seen that the formulation F2 is ideal; thus, it was chosen for further characterization, such as texture analysis.

### 2.10. Gel Strength Analysis

The development of items, such as gels, capsules, and contact lenses, depends on the strength, flexibility, and rupture force of the items. The ability of a colloidal dispersion to produce and maintain a gel form is gauged by its gel strength. Bloom is the conventional name for gel strength in the gelatin industry. It indicates the amount of force, measured in grams, required to compress a gelatin gel’s surface by 4 mm when using a cylinder probe with a standard 0.5-inch diameter. It is the measure of force required to rupture a gel. The bloom strength of the prepared polyherbal gel is 0.0163 kg, as shown in Table 8 and Figure S1 (Supporting Information). This property influences the penetration of the drug from the gel matrix [19].

### 2.11. Extrudability

This mechanical property plays a vital role in the selection, packing, and removal of a gel from its container. To assess how easily topical preparations, such as ointments, creams, and gels, can be removed and applied, extrudability must be quantified. The consistency of a product can change over the course of its shelf life, and product developers can analyze these changes and modify formulations accordingly. This allows producers to evaluate the compatibility of a packaging material and its design. Rheological property also influences the spreadability, firmness, and in vivo performance of a product upon its application to the skin [20]. The results corresponding to extrudability are shown in Table 9 and Figure S2 (supporting information). The results indicate that the gel requires 3.401 kg force to extrude through the outlet. Therefore, the formulated gel has ideal extrudability.

## 3. Conclusions

The development of polyherbal formulations has drawn increasing attention due to its historical roots, economic viability, and patient compliance. The preliminary assessment and antimicrobial study of *Vigna radiata* demonstrated a strong antimicrobial effect of the extract against acne infection. Gels are becoming more and more popular. Compared to other semisolid preparations, including ointments, creams, pastes, etc., they can give controlled release and are more stable. Making gels can result in improved absorption, which increases medicinal drugs’ bioavailability. Gels’ long-term stability features open up possibilities for their beneficial application to patients. Gels are simple to make, but extensive drug and excipient modification is required to produce a stable, effective, and secure product. The polyherbal gel formulated in this study indicates that it might be a good gel for topical application that has the additive effects of *Aloe barbadensis*. Additionally, the exact mechanism of action of the gel on the skin can be explored through extensive pharmacological tools at the molecular level so that it would be an effective way to use in a rational way. Further studies to characterize the pharmacokinetics of the polyherbal gel and to establish its safety, stability, and anti-acne activity will be investigated using various new formulations at varied strengths and dosage forms, as well as with different plant extracts.

## 4. Materials and Methods

### 4.1. Materials

*Aloe barbadensis* was collected from the medicinal garden of Karpagam College of Pharmacy, and the seeds of *Vigna radiata* were procured from a local market and were authenticated by the Department of Botanical Survey, TNAU, Coimbatore. All other materials used were of analytical grade and bought from HiMedia Laboratories Private Ltd., Mumbai, India.

### 4.2. Methods

#### 4.2.1. Extraction of *Vigna radiata*

The seeds of *Vigna radiata* were collected and milled into fine particles. About 500 g of the crushed *Vigna radiata* powder was extracted by the Soxhlation process using ethanol as a solvent. The process continued till the solvent turned out to be clear. The extract was evaporated to dryness using a desiccator [21].

#### 4.2.2. Collection of *Aloe barbadensis* Gel

Fresh leaves of *Aloe barbadensis* were collected. The outer thick epidermis of the leaf was selectively removed, and the inner gel-like pulp in the center of the leaf was separated, minced, and homogenized in a mortar and pestle. It was filtered using a muslin cloth to obtain a clear liquid [22].

#### 4.2.3. Evaluation of the *Vigna radiata* Extract

##### Characteristics of Extract

The ethanolic extract of the *Vigna radiata* was evaluated for its physical state, color, and odor.

##### Phytochemical Investigation of the Extract

The alcoholic extract of *Vigna radiata* was screened qualitatively for the presence of various phytoconstituents, such as flavonoids, proteins, amino acids, phenol, and organic acid.

##### Determination of Anti-Infective Activity

The anti-acne activity of the extract against infectious agents were performed using the standard cultures of *Staphylococcus aureus* (ATCC-6538P), *Escherichia coli* (ATCC-8739), and *Candida albicans* (ATCC-18804).

#### 4.2.4. Standardization of Inoculum

The inoculum was standardized using the serial dilution agar-plate method by aseptically transferring 1 mL of the bacterial suspension tube into sterile water of a predetermined volume, where the culture was diluted 10 times from 10^–1^ to 10^–10^. The diluted suspensions were gently rotated as they were put into a Mueller–Hinton agar medium incubated for 24 h at 37 °C, and then counted using a colony counter. By multiplying the cells seen on each plate by the dilution factor, the number of colonies contained in the sample was determined [23].

#### 4.2.5. Antimicrobial Activity by Cup Plate Method

The sterile Petri dishes were filled with a Mueller–Hinton agar medium and then infected with the test organisms at the appropriate dilution (*Staphylococcus aureus*, *Escherichia coli*, and *Candida albicans*). In the medium, four cylinders or cups were created using a sterile borer on each plate. A consistent amount of 0.2 mL of the solution was poured into the cup, which was then incubated for 24 h at 37 °C. Three duplicates of the investigation were conducted, and the results were reported as mean inhibition in diameter (mm) [24].

#### 4.2.6. Formulation of Gel Base and Optimization

The gelling agent, Carbopol 940, at various concentrations (Table 4) were dispersed in an adequate quantity of water. Propylene glycol used as a humectant or plasticizer was also added. The preservatives, methylparaben and propyl paraben, were transferred and blended. The pH was altered to neutral by the addition of triethanolamine, and the final weight of the gel was made up to 50 g with distilled water. The above mixture was stirred for 2 h at 500 rpm to become void of air bubbles and kept at room temperature for 24 h to observe its stability and consistency [25].

#### 4.2.7. Formulation of Polyherbal Gel Containing *Vigna radiata* and *Aloe barbadensis*

The polyherbal gel was prepared by incorporating the ethanolic extract of *Vigna radiata* at various concentrations and 5 mL of *Aloe barbadensis* liquid into the optimized Carbopol gel as per the formula mentioned in Table 5. The entire mass was stirred at 500 rpm for 2 h and kept undisturbed for a day at room temperature [26].

#### 4.2.8. Physical Characterization of the Formulated Acne Gel

The polyherbal gel formulation was subjected to physical characterization, such as color, appearance, pH, viscosity, and spreadability.

#### 4.2.9. Physical Appearance

The formulated gel was inspected for its organoleptic characteristics, viscosity, and homogeneity after being packed in the container and verified for the appearance and existence of any aggregates.

#### 4.2.10. Determination of pH

About 1 g of the gel was mixed in 100 mL of deionized water. The determination of pH of individual formulation was determined using a digital pH meter (Model MK–VI, Kolkata, India) carried out three times to obtain triplicate readings.

#### 4.2.11. Determination of Viscosity

The viscosity of the formulated gel was performed in a cup-and-bob type of rotational viscometer (Brookfield viscometer RVT) with spindle No.62.

#### 4.2.12. Spreadability

The spreadability of the gel was calculated to anticipate how much of an area it would spread when applied to skin. A thin coating of 100 g of the gel was applied between two slides, which had a 6 cm border around them. The slides were then fastened to an undisturbed platform in such a way that only the upper slide could be released freely by the weight that was tied to it. A 20 g mass was attached to the upper slide. The amount of time it took for the upper slide to move a predetermined distance before being torn apart by the impact was noted [19]. Triplicates were carried out.

#### 4.2.13. Analysis of Gel Strength and Extrudability

The formulated gel was analyzed for its strength using a calibrated texture analyzer TA-XT2. Initially, the instrument was calibrated for force and distance measurement at room temperature. The 45° cap was partly filled with 1 g of the gel and set on the platform of the analyzer. A corresponding 45° cone was used as a probe to spread and detect the dynamics of spreading and retracting forces as it moved vertically toward the bottom of the cap followed by withdrawal to its original point. The cone and cap assembly were aligned coaxially. During the test, the cone probe traveled downward at a speed of 3 mm/s until it reached a distance of 1 mm from the bottom of the cap. This was immediately followed by an upward movement of the probe (i.e., retraction mode) at a speed of 10 mm/s. The total work performed to spread 1 g of the gel in between the cone–cap surfaces and the total work required to retract from the spread gel represent the gel strength [27].

Extrudability was measured using the HDP/FE forward extrusion cell of the TA-XT2 texture analyzer (UK). The compression force is the force required for a piston disc to extrude a product through a standard-sized outlet in the base of the sample container. The sample was placed in a centralizing insert filled into a heavy-duty platform, and a piston disc was attached to a load cell (5000 g) using a probe adopter. The plunger compressed the sample and caused forward flow through the annulus of the disc. The compression force sensed by the load cell represents the extrudability [28].

## Figures and Tables

**Table 1:** Evaluation of organoleptic characteristics of the extract.

Characteristics	Observation
Physical state	Semisolid
Color	Green
Odor	Characteristic
Taste	Characteristic

**Table 2:** Screening of phytochemical constituents of *Vigna radiata*.

Constituents	Test	End Point	Results
Flavonoids	Ferric chloride	Green color	++
	Lead acetate	Yellow precipitate	++
Protein	Xanthoprotein	Yellow precipitate	++
	Ninhydrin	Blue color	++
Amino acids	Ninhydrin	Purple or bluish color	++
	Tyrosine	Dark red color	++
Phenol	Ferric chloride	Blue or red color	++
Organic acid	Phosphoric acid	Light yellow Precipitate	++

++ Presence of active constituents.

**Table 3:** Antimicrobial activity of the extract of *Vigna radiata*.

Sample	*Staphylococcus aureus* (ATTC-6538P)	*Escherichia coli* (ATCC-8739)	*Candida albicans* (ATCC-18804)
		Inhibitory Zone (mm)	
Polyherbal gel (F2)	12.3 ± 0.1	13.2 ± 0.06	12.4 ± 0.1
Gentamicin (10 mcg)	22.2 ± 0.06	22.3 ± 0.1	-
Fluconazole (25 mcg)	-	-	22.2 ± 0.03

**Table 4:** Formulation of Carbopol gel.

Ingredients	G1	G2	G3
Carbopol 940	1%	1.5%	2%
Propylene glycol	5 mL	5 mL	5 mL
Methyl paraben	0.15 g	0.15 g	0.15 g
Propyl paraben	0.30 g	0.30 g	0.30 g
Triethanolamine	5 mL	5 mL	5 mL
Water	q. s	q. s	q. s

**Table 5:** Formulation of polyherbal gel.

Ingredients	F1	F2	F3
*Vigna radiata* extract	1%	1.5%	2%
*Aloe barbadensis* gel	5 mL	5 mL	5 mL
Carbopol 940	2%	2%	2%
Propylene glycol	5 mL	5 mL	5 mL
Methyl paraben	0.15 g	0.15 g	0.15 g
Propyl paraben	0.30 g	0.30 g	0.30 g
Triethanolamine	5 mL	5 mL	5 mL
Water	q. s	q. s	q. s

**Table 6:** Physical appearance of the formulated gel.

Characteristics	F1	F2	F3
**Physical appearance**	Transparent gel	Transparent gel	Transparent gel
**Color**	Pale yellow	Pale yellow	Pale yellow
**Homogeneity**	Absence of aggregates	Absence of aggregates	Slight aggregates

**Table 7:** Measurement of pH, viscosity, and spreadability.

Formulation Code	pH	Viscosity (cps)	Spreadability (g cm/s)
F1	5.9	1428 ± 0.1	19.37
F2	5.7	1425 ± 0.8	21.35
F3	5.8	1358 ± 0.3	22.13

**Table 8:** Test data of gel strength.

Gel Strength/ m Value (g) Force 1	Force at Target (cycle:1) (kg)	Radiant to Positive Peak (cycle:1) kg/s
0.693	0.016	0.004

**Table 9:** Test data of extrudability.

Firmness (g)	Force at Target (Cycle:1) (kg)	Force at 5 mm (Cycle:1) (kg)
3168.854	3.369	3.228

## Data Availability

All data are available in the manuscript.

## Supplementary Materials

The following supporting information can be downloaded at https://www.mdpi.com/article/10.3390/gels9020163/s1. Figure S1: Graphical representation of gel strength; Figure S2: Graphical representation of extrudability.
