# Supplementary material for: Development of a Polyherbal Topical Gel for the Treatment of Acne
Source: Gels. 2023 Feb 17;9(2):163. doi: 10.3390/gels9020163 (PMC9956052; doi:10.3390/gels9020163)
Supplement: Supplementary file 1 [file gels-09-00163-s001.zip › gels-2138817-supplementary.pdf]

gels

MDPI

Article

Development of a Polyherbal Topical Gel for the Treatment of Acne

Benedict Jose Chellathurai [1,*], Ramyadevi Anburose [1], Mohammad H. Alyami [2,*], Mohan Sellappan [1], Mohammad F. Bayan [3], Balakumar Chandrasekaran [3], Kumarappan Chidambaram [4] and Mohamed Rahamathulla [5]

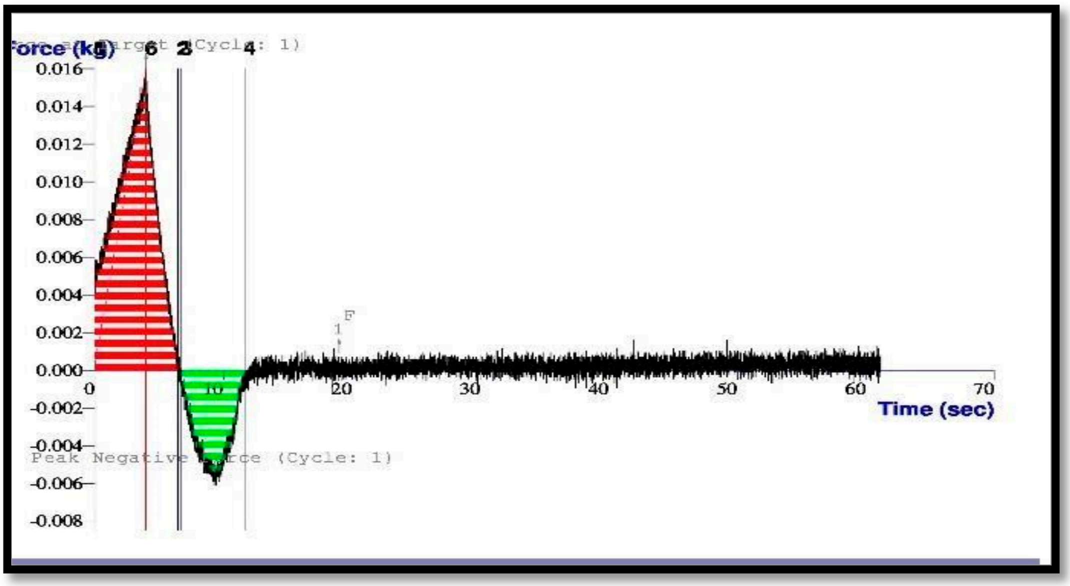

Figure S1. Graphical representation of gel strength.

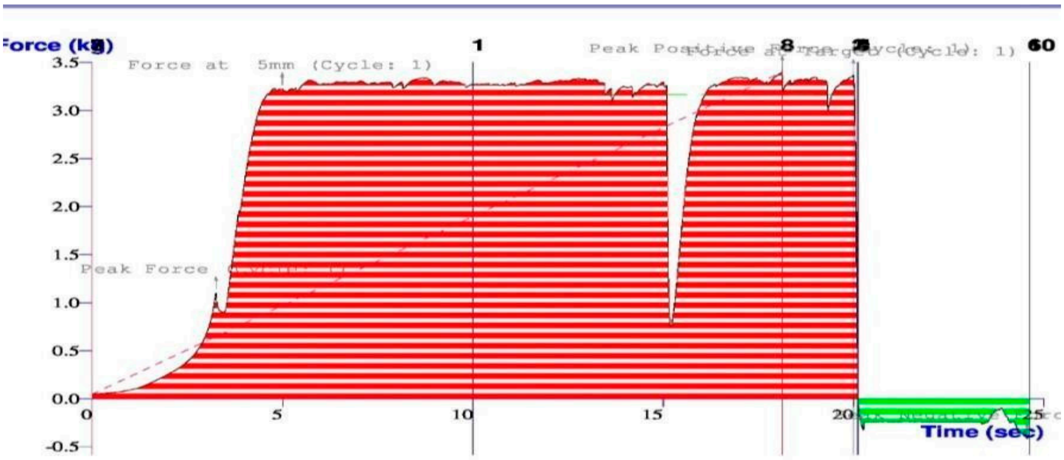

Figure S2. Graphical representation of extrudability.
